# A Rare Cause of Recurrent Oral Lesions: Chediak-Higashi Syndrome

**DOI:** 10.4274/tjh.2013.0282

**Published:** 2014-09-05

**Authors:** Müsemma Karabel, Selvi Kelekçi, Velat Şen, Duran Karabel, Çiğdem Aliosmanoğlu, Murat Söker

**Affiliations:** 1 Dicle University Faculty of Medicine, Department of Pediatrics, Diyarbakır, Turkey; 2 Dicle University Faculty of Medicine, Department of Pediatrics, Division of Hematology Oncology, Diyarbakır, Turkey

**Keywords:** child, Chediak-Higashi syndrome, Oral lesions

## TO THE EDITOR

Chediak-Higashi syndrome (CHS) is a rare autosomal recessive disorder characterized by oculocutaneous albinism, recurrent bacterial infections, and giant granules in peripheral blood leukocytes or bone marrow cells. There are 2 phases of the disease. The first is the stable phase, which is characterized by recurrent infections such as periodontitis and gingivitis due to neutropenia and/or neutrophil dysfunction and hypopigmentation of the hair, skin, and eyes. It typically develops in infancy or early childhood in most patients with CHS. The second phase is the accelerated phase, which is characterized by persistent fever, hepatosplenomegaly, lymphadenopathy, pancytopenia, and bone marrow infiltration and hemophagocytosis by histiocytes, similar to findings of hemophagocytic syndrome (HLH) [1,2,3,4]. In this letter, we report a very rare presentation of CHS in an 11-year-old girl who was in the accelerated phase of the syndrome and presented with recurrent oral ulcers and periodontitis when she was 1 year old.

**Figure 1:**
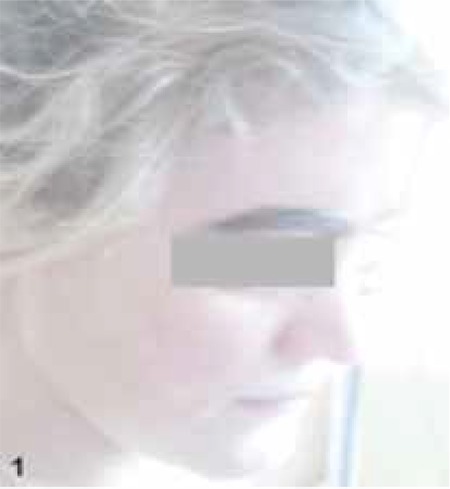
Decreased pigmentation in hair and skin.

An 11-year-old girl with complaints of recurrent oral ulcers for 10 years and persistent fever, abdominal pain, and fatigue for 2 weeks was admitted to our clinic. She also had a history of photophobia with a gradual decrease in vision, generalized weakness, and weight loss over the past few years. Physical examination revealed a pale, lethargic child with mental retardation and developmental delay. She had oral cavity ulcers, gingivitis, periodontitis, silvery hair, whitish eyebrows and eyelashes, and hypopigmented and hyperpigmented spots on areas of the skin exposed to the sun (Figure 1). She had also hepatosplenomegaly, lymphadenopathy, and neurological abnormalities such as decreased deep tendon reflexes and muscle weakness in the lower extremities.

**Figure 2:**
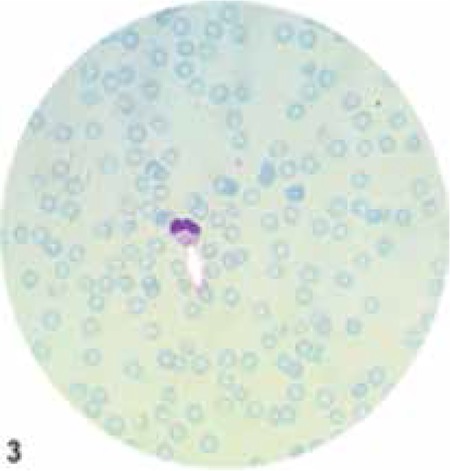
A neutrophil showing giant granules.

Laboratory investigations showed pancytopenia with hemoglobin concentration of 7.3 g/dL, platelet count of 57x109/L, white blood cell count of 1.99x109/L, absolute neutrophil count of 157/L, prothrombin time-international normalized ratio of 1.28, activated partial thromboplastin time of 45 s, and lactate dehydrogenase level of 1122 U/L (normal range: 0-250). The levels of serum triglyceride and total/direct bilirubin levels were 378 mg/dL and 1.7/1.1 mg/dL, respectively. Alanine and aspartate transaminase levels were 180 and 200 IU/L, respectively. Serum ferritin and plasma fibrinogen levels were 586 µg/L and 120 mg/dL, respectively. Total immunoglobulin G levels were found to be elevated for the patient’s age (2650 mg/dL). In the peripheral blood smear, the presence of several abnormal giant inclusion bodies in the neutrophils (Figure 2) and single large inclusion bodies in most lymphocytes was observed. Bone marrow smear showed giant granules in the cytoplasm of the myeloid and monocytic cell series and revealed no blasts or hemophagocytoses. Although hemophagocytosis could not be demonstrated, the patient fulfilled the diagnostic criteria of HLH [5]. Thus, the accelerated phase of CHS was diagnosed on the basis of the clinical presentation and hematologic findings. The patient was treated with appropriate antibiotics and replacement therapy. She is currently under observation with continued symptomatic treatment and awaiting bone marrow transplantation. Informed consent was obtained.

The early diagnosis of recurrent oral lesions in CHS, particularly in relation with the stable phase, is still unclear. Although recurrent infections, developmental delay, and progressive muscle weakness were present in our case since 1 year of age, it was striking that the main complaint of the patient was recurrent aphthous lesions in the mouth and sore gums. The patient had oral lesions since 1 year of age but was diagnosed in the accelerated phase of CHS at 11 years of age, which was very late compared to other patients with recurrent oral lesions [3,6]. Rezaei et al. [6] reported recurrent oral ulcers in 2/5 cases of CH1S with moderate neutropenia.

The diagnosis of CHS should be considered in any child with recurrent unexplained gingivitis and periodontitis along with hypopigmentation of the hair, skin, and eyes, as in the presented case. These findings should alert physicians to the need to investigate giant granules in peripheral blood and bone marrow smears. Finally, early diagnosis and treatment of CHS in the stable phase is very important to prevent severe hematological and neurological complications later.

## CONFLICT OF INTEREST STATEMENT

The authors of this paper have no conflicts of interest, including specific financial interests, relationships, and/ or affiliations relevant to the subject matter or materials included.
